# When to Start and Stop Bone-Protecting Medication for Preventing Glucocorticoid-Induced Osteoporosis

**DOI:** 10.3389/fendo.2021.782118

**Published:** 2021-12-15

**Authors:** Kaleen N. Hayes, Ulrike Baschant, Barbara Hauser, Andrea M. Burden, Elizabeth M. Winter

**Affiliations:** ^1^ Department of Health Services, Policy, and Practice, Brown University School of Public Health, Providence, RI, United States; ^2^ Division of Endocrinology, Diabetes and Bone Diseases, Department of Medicine III and Center for Healthy Aging, Technische Universität Dresden, Dresden, Germany; ^3^ Rheumatic Disease Unit, Western General Hospital, National Health Service (NHS) Lothian, Edinburgh, United Kingdom; ^4^ Rheumatology and Bone Disease Unit, Centre for Genomic and Experimental Medicine, Institute of Genetics and Molecular Medicine, University of Edinburgh, Edinburgh, United Kingdom; ^5^ Institute of Pharmaceutical Sciences, Department of Chemistry and Applied Biosciences, Swiss Federal Institute of Technology [Eidgenössische Technische Hochschule (ETH)] Zurich, Zurich, Switzerland; ^6^ Center for Bone Quality, Division of Endocrinology, Department of Internal Medicine, Leiden University Medical Center, Leiden, Netherlands

**Keywords:** glucocorticoid-induced osteoporosis, glucocorticoids, bone fractures, bone density, anti-resorptive treatment, bone density conservation agents, bisphosphonates, teriparatide

## Abstract

Glucocorticoid-induced osteoporosis (GIOP) leads to fractures in up to 40% of patients with chronic glucocorticoid (GC) therapy when left untreated. GCs rapidly increase fracture risk, and thus many patients with anticipated chronic GC exposures should start anti-osteoporosis pharmacotherapy to prevent fractures. In addition to low awareness of the need for anti-osteoporosis therapy among clinicians treating patients with GCs, a major barrier to prevention of fractures from GIOP is a lack of clear guideline recommendations on when to start and stop anti-osteoporosis treatment in patients with GC use. The aim of this narrative review is to summarize current evidence and provide considerations for the duration of anti-osteoporosis treatment in patients taking GCs based on pre-clinical, clinical, epidemiologic, and pharmacologic evidence. We review the pathophysiology of GIOP, outline current guideline recommendations on initiating and stopping anti-osteoporosis therapy for GIOP, and present considerations for the duration of anti-osteoporosis treatment based on existing evidence. In each section, we illustrate major points through a patient case example. Finally, we conclude with proposed areas for future research and emerging areas of interest related to GIOP clinical management.

## Introduction

Glucocorticoids (GCs) are potent immunosuppressive and anti-inflammatory medications with a host of beneficial and negative effects (1). GCs are commonly prescribed to reduce inflammation and to suppress the immune system for a broad spectrum of indications, including chronic lung disease, inflammatory arthritis, connective tissue disease, and organ transplantation. As such, GC use is prevalent globally: at any time, 1.0 to 4.6% of UK and US adults (up to 13.7 million persons, collectively) are taking oral GCs, and 27% to 65% of these patients will receive long-term (≥3 months) GC treatment (2–6). GCs are essential medications, but chronic GC use has detrimental effects, including metabolic disorders [e.g., type 2 diabetes mellitus (7)]; impaired wound healing (8); increased risk of infection (9); and glucocorticoid-induced osteoporosis (GIOP), the most common cause of secondary osteoporosis (10). GCs effects on bone health are potent, and they increase fracture risk independently of other risk factors like low bone mineral density (BMD) (11). Untreated GIOP can lead to debilitating fractures that cause morbidity, with reduced quality-of-life, mortality, and healthcare costs (12–14). Up to 40% of patients who have GC use longer than 3 months will experience a vertebral fracture (15). Thus, anti-osteoporosis treatment is indicated for patients on long-term GC therapy to preserve bone health and reduce fracture risk. Many pharmacologic therapies for primary osteoporosis, like antiresorptive treatments and teriparatide, have evidence of anti-fracture benefits in patients with GIOP (16–18).

Unfortunately, GIOP is underdiagnosed and undertreated (6, 19–23). In one population-based US study from 2006, only half of all postmenopausal women with long-term GC use received anti-osteoporosis treatment, and this proportion decreased to 5% for women less than 50 years of age and men (6). A more recent investigation found only 42% of US patients with chronic conditions warranting glucocorticoid exposure received any osteoporosis monitoring or treatment (24). Lack of awareness of the fracture risk caused by GC use limits appropriate initiation of anti-osteoporosis therapy (25, 26). In addition, an urgent focus on management of the condition for which GCs are prescribed (e.g., active rheumatoid arthritis [RA]), which may include a plethora of tests and examinations to assist in diagnosis and symptom improvement, may also contribute to poor anti-osteoporosis treatment levels. Fortunately, some interventions have shown to substantially improve uptake of therapy to prevent GIOP and fractures: a recent educational program in the UK improved the proportion of patients on chronic GCs who were indicated for therapy from 25 to 92% (27). Other educational interventions have improved treatment, yet to a lesser degree (28). However, even among clinicians aware of the risk of GIOP, appropriate treatment is largely hindered by a lack of clear evidence and recommendations regarding populations that are indicated for GIOP therapy and when to start and stop treatment to prevent GC-induced fractures.

**Figure 1:**
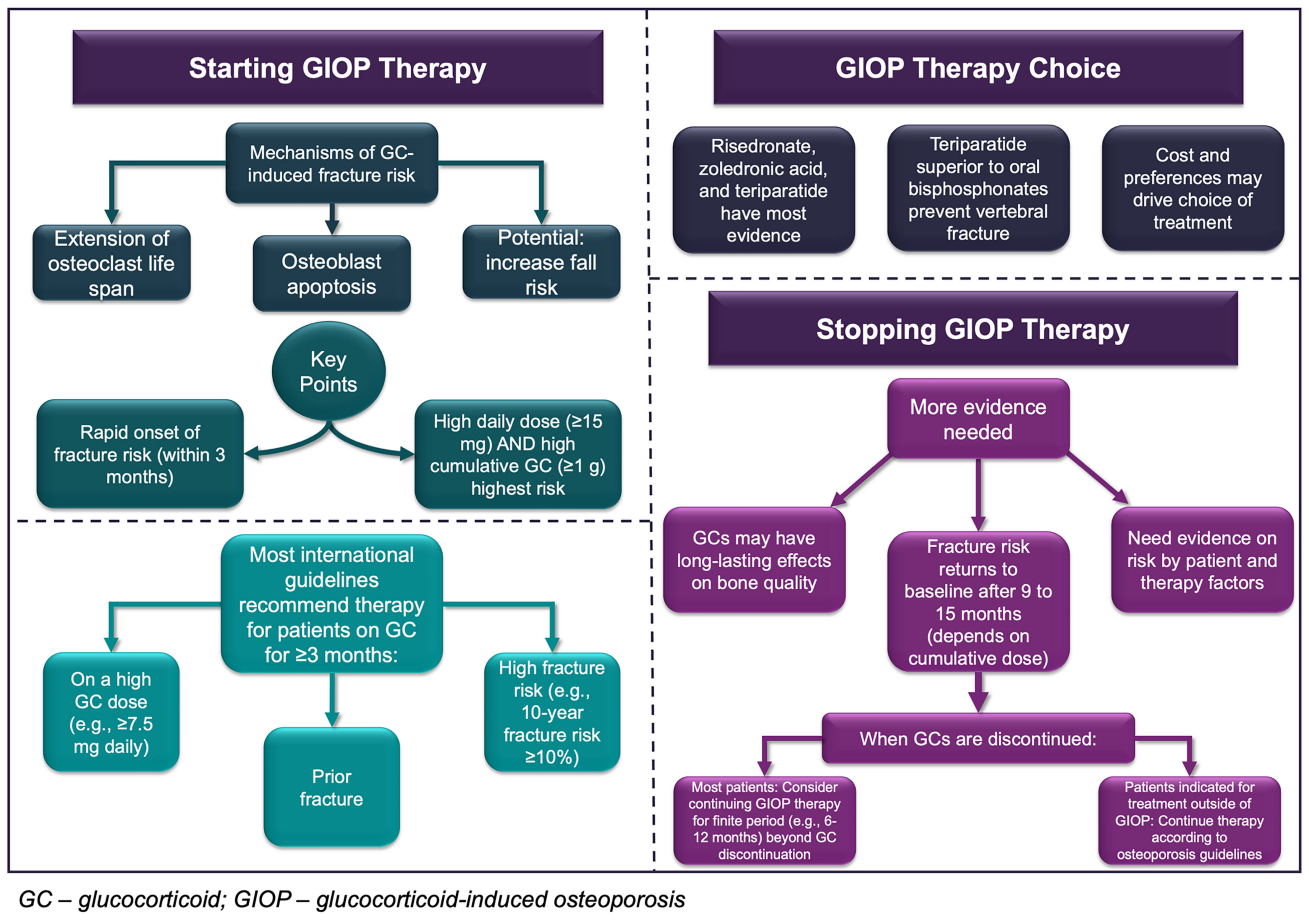
Graphical representation of the main points presented in this article.

The aim of this narrative review is to summarize current evidence and provide considerations for the initiation and discontinuation of anti-osteoporosis therapy for patients taking systemic GCs based on pre-clinical, clinical, epidemiologic, and pharmacologic evidence. Inhaled GCs or GC replacement (i.e., Addison’s disease) are not considered in this review. We first provide an overview of GIOP pathophysiology, review current guideline recommendations for anti-osteoporosis therapy initiation among patients with long-term GC use, and present considerations regarding the discontinuation of anti-osteoporosis treatment for GIOP. We use a mock patient case to illustrate the key points and clinical debates that exist throughout the review. We then conclude with proposed areas for future research and emerging topics of interest related to GIOP clinical management. The main points of this article are presented graphically in **Figure 1**.

## Pathophysiology and Epidemiology of GIOP

### Patient Case: Part 1

Your patient is a 45-year-old Caucasian pre-menopausal female (pronouns: she/her) living in Canada who has been recently diagnosed with systemic lupus erythematosus (SLE) with musculoskeletal and mucocutaneous involvement and a SLE Disease Activity Index (SLEDAI) of 6, indicating moderate disease activity (29). You initiate hydroxychloroquine (HCQ) therapy and a tapering course of prednisone 15 mg per day, reduced by 2.5 mg weekly down to 2.5 mg prednisone per day. A 6-week follow-up visit is scheduled. Your patient has no other chronic conditions or prior fractures, does not smoke, consumes 2 alcoholic drinks per week, and has a BMI of 23.

Should you initiate anti-osteoporosis therapy for this patient at her initial appointment? Do you need additional investigations (e.g., dual-energy X-ray absorptiometry [DXA] scan)?

### GIOP Pathophysiology

GCs increase fracture risk through a variety of mechanisms. Like the pathogenesis of primary osteoporosis, GCs induce an imbalance in the bone remodeling cycle governed by osteoclasts and osteoblasts that break down and build bone, respectively (30). Bone loss from GC exposure occurs in two major time periods: a rapid initial phase where approximately 2-9% of the BMD is lost within the first six months depending on GC dose, with a steady reduction in BMD of about 0.5% to 2% annually during continued treatment (11, 31–35). GCs decrease BMD in trabecular bone, mainly in the vertebrae and femoral neck, to a greater extent than in other types of bone (11, 36). Initially, GCs appear to induce a transient excess of bone resorption. GCs extend the lifespan of osteoclasts through upregulation of receptor activator nuclear factor kappa-B ligand (RANKL) while suppressing osteoprotegerin (OPG) in osteogenic cells and suppression of apoptosis signals (37, 38). Thereafter, GCs cause their most profound negative effects on bone formation and quality (39) by reducing pro-osteogenic gene expression and suppressing osteoblast differentiation and proliferation, inducing apoptosis in osteoblasts and osteocytes, and increasing osteocyte autophagy (40). GCs also disrupt the function of bone marrow stromal cells (41), preventing their subsequent maturation to osteoblasts and osteocytes (42). Comprehensive reviews with further details of *in vitro* and *in vivo* studies of glucocorticoid effects on bone cell function have been recently published (39, 43).

GCs appear to increase fracture risk beyond their effects on bone turnover and BMD. The independence of fracture risk from BMD changes is even more profound for GIOP than primary osteoporosis. Multiple randomized controlled trials (RCTs) have demonstrated that patients taking GCs had a significantly higher vertebral fracture risk compared to similar patients with primary osteoporosis and the same BMD values (31, 32, 44, 45). A meta-analysis of epidemiologic studies showed that the BMD changes seen at the spine and hip among GC users would correlate to an expected relative risk of vertebral and hip fracture of 1.48 and 1.41, respectively, in patients with primary osteoporosis (45); however, the observed relative risks for vertebral and hip fracture were 2.40-3.05 and 1.54-2.34, respectively, depending on the patient’s cumulative GC exposure (11). To account for the poorer correlation between BMD and fracture risk in GIOP, 10-year fracture probabilities from the fracture risk assessment (FRAX) tool are often increased by a factor of 1.15 to 1.20 for patients currently exposed to 7.5 or more milligrams (mg) of prednisone equivalents per day (prednisone equivalent daily dose; henceforth, DD) (46). The disparity between the predicted and observed fracture risk in patients taking GCs compared to primary osteoporosis is often attributed to GC effects on bone quality, though mechanisms for this effect are unclear. The extension of osteoclast cell lifespans by GCs may impair osteoclast functioning long-term (47), reducing rates of bone turnover and potentially resulting in lower bone quality. GCs also affect the mineralization of bone by reducing expression of bone matrix proteins, and GC effects on osteoblasts likely reduce bone quality as well (30).

### Fracture Risk Associated With GC Use: Effects of Daily and Cumulative Dose

GC use strongly increases fracture risk, with highest observed effects on vertebral fractures (48). Compared to matched controls, patients on any dose of long-term GC therapy have an average 3-times higher risk of vertebral fracture and a 2-times higher risk of hip fractures (11). As in primary osteoporosis, vertebral fractures can be asymptomatic and not come to clinical attention. Up to 40% of patients taking chronic GCs have an asymptomatic vertebral fracture, and 14% have two or more asymptomatic fractures (15).

Dose-dependent effects of GCs on bone are also well-established. A large observational UK cohort study found that hip fracture risk was 2.21-times higher among those taking 7.5 mg or more per day versus GC users taking less than 2.5 mg per day (11). This dose-dependent relationship was even more profound for vertebral fracture, where the risk among those taking 7.5 mg or more per day was 2.83-times higher compared to those taking less than 2.5 mg per day. Even low-dose chronic GC therapy (<2.5 mg per day DD) is associated with a 1.5-times increased vertebral fracture risk compared to no use (11). Low doses do not appear to affect hip fracture risk (relative rate 0.99 [95% CI 0.85 to 1.20]) (11). A more recent study confirmed that, after confounder adjustment, GC doses below 7.5 mg per day DD independently increased the risk of clinical vertebral fractures (HR 1.59 [95% CI 1.11-2.29]) with strong dose-response effects, but no association with overall osteoporotic fracture risk was found (49).

Cumulative GC exposure also appears to affect fracture risk independently of the DD. A case control study of Danish data found that, compared to never-users, a high DD (≥15 mg) and high cumulative dose (≥1 gram prednisone equivalent cumulative dose [CD]) were independently associated with hip fractures (adjusted odds ratio [OR_adjusted_] for DD ≥15 mg: 1.64 [95% CI 1.54 -1.74]; OR_adjusted_ for CD ≥1 gram: 2.50 2.19-2.85)] and clinical vertebral fractures (OR_adjusted_ for DD ≥15 mg: 3.75 [95% CI 2.97 to 4.77]); OR_adjusted_ for CD ≥1 gram: 2.57 [95% CI 2.30 to 2.87]) (50). Those with both high DD and high CD were at greatest risk (DD ≥15 mg and CD ≥1 gram: OR_adjusted_ for clinical vertebral fracture 4.36 [95% CI 3.32-5.72] and hip fracture OR_adjusted_ 2.94 [95% CI 2.52-3.43]) (50). Another study found that intermittent high-dose GC without high cumulative exposure (≥15 mg/day DD but <1 gram total CD) was associated with a modest increased risk in vertebral fracture and no other fracture risk, but the risk of all types of fracture increased dramatically if the patient had cumulative GC exposures ≥1 gram (51). Similarly, another population-based Danish case-control study illustrated that among patients with COPD, intermittent high dose GC use (≥15 mg DD) was only associated with osteoporotic fracture risk when the CD exceeded 1 gram (52). In a population-based US study, a subgroup of patients less than 50 years of age only experienced a higher risk of fracture after receiving a CD of 1350 mg or higher (24). Conversely, in the CPRD study, the association between CD and fracture was nullified after accounting for the DD, age, and other potential confounders (11). However, a high DD and a CD greater than 5 grams was associated with a profound increase in all fracture risk compared to prior periods of no exposure (vertebral fractures: RR 14.42 [95% CI 8.29 to 25.08]; hip fractures: RR 3.13 [95% CI 1.49 to 6.59]) (11).

GCs may also indirectly increase fracture risk through other mechanisms. GCs induce muscle atrophy by reducing protein synthesis (30). Decreased muscle strength and insufficient balance can thus lead to falls and increase impact of a fall, particularly in older adults (53–55). GCs also impair gastrointestinal and renal reabsorption of calcium and may result in hypocalcemia and subsequent disruption of the bone turnover cycle (56), though evidence on whether these changes have clinical impacts remains controversial (57). Finally, some diseases that GCs are used to manage and treat (e.g., RA) have detrimental effects on bone from chronic inflammation (58, 59). It is still uncertain whether there is a tolerable dose of GCs for those with severe conditions that may prevent disease-induced bone loss while avoiding increasing risk through the aforementioned mechanisms either through low doses, intermittent use, or concurrent use of anti-osteoporosis therapies (59). For example, two prospective studies in SLE patients showed that a DD of less than 7.5 mg was not associated with bone loss in these patients (60, 61); however, these low GC doses have been consistently demonstrated to increase vertebral fracture risk in other populations, so this topic remains controversial (11, 49).

### Patient Case: Part 1 Response

At her initial appointment, anti-osteoporosis treatment is not indicated for MP, and no DXA scan is needed at this stage. First, the target prednisone DD (2.5 mg) and estimated CD after 6 weeks (367.5 mg) are below thresholds where fracture risk increases in patients younger than 50 years of age (7.5 mg DD per day and 1-1.350 g total CD) (11, 24, 49). Next, as GC treatment is planned to be used as bridging therapy until HCQ is anticipated to take effect, we can anticipate that MP will receive fewer than 3 months of GC exposure and therefore is at lower risk of fracture (11). Finally, outside of her GC exposure, due to young age and no other major fracture risk factors (e.g., no recent fragility fracture or prior vertebral fractures), she has an overall low fracture risk (FRAX score estimating a 4.5% risk of sustaining a major osteoporotic fracture over the next 10 years).

## Starting Treatment to Prevent GIOP

### Patient Case: Part 2

At her 6-week follow-up appointment, your patient reports that her joint symptoms have worsened. She has ongoing mouth ulcers and she reports pleuritic chest pain. In addition, she is found to have proteinuria, low complement levels, and elevated double-stranded DNA antibody levels resulting in a high SLEDAI score (16). You decide to initiate azathioprine as adjunct treatment to HCQ and increase her prednisone dose to 40 mg daily for 1 month with subsequent reduction to a maintenance dose of 10 mg daily thereafter until her symptoms are better managed and disease score reduced. The patient also undergoes a DXA scan, and her femoral neck BMD T-score is -1.6. Her updated calculated FRAX score suggests a 6.1% probability of sustaining a major osteoporotic fracture in the next 10 years.

Should you initiate anti-osteoporosis therapy at this visit?

### GIOP-induced Fracture Risk at Treatment Onset

Evidence suggests that bone loss and fracture risk increases rapidly following GC initiation (62). A meta-analysis of ten observational studies showed that the largest decrease in BMD occurs in the first three months of GC treatment among first-time users, regardless of daily dose (11). When considering fracture risk, RCTs have shown an elevated vertebral fracture risk in the first year after GC therapy initiation (21–25), while population-based studies have found that fracture risk occurs within three to six months after initiation (8, 20). One observational study demonstrated a heightened fracture risk in the first 30 days after GC initiation among adult patients less than 65 years of age using a self-controlled case series design (62). The exact onset of fracture risk may also differ between patients with varying baseline fracture risk.

While fracture risk remains heightened in users of GCs throughout treatment, both fracture risk and the rate of bone loss appear to stabilize after the first six to 12 months of exposure, even among those receiving high GC doses (2, 11). This pattern of risk may be due to the biphasic effects of GCs on bone. The rapid increase in fracture risk in the first months of GC exposure likely results from the initial rapid increase in bone resorption due to enhanced osteoclast activity that results in a negative uncoupling between bone formation and bone resorption phases (63). This early phase is paralleled by a second, more progressive phase where bone formation is hampered (40, 64). Longitudinal gene expression profile studies have indeed shown an early induction of genes related to osteoclast function followed by a long-lasting suppression of genes related to osteoblasts (65). Thus, the sustained, but stable, fracture risk after the initial period likely results from the long-term effects of glucocorticoids on osteoblast proliferation and function.

### Current Treatments for GIOP

Current therapies approved for the treatment of glucocorticoid-induced osteoporosis in most jurisdictions include oral bisphosphonates (21–23); intravenous bisphosphonates (24), primarily zoledronic acid; denosumab (66–68); and anabolic agents (18), primarily teriparatide (44, 69). Most therapies were approved to treat GIOP primarily on the basis of BMD bridging studies (70), with larger trials suggesting anti-fracture effectiveness thereafter (16, 17, 69). Oral bisphosphonates have been shown in multiple trials and population-based studies to be associated with a significantly reduced fracture risk in GC users (e.g., HR 0.58 [95% CI 0.51 to 0.66] for vertebral fractures and HR 0.71 [95% CI 0.57 to 0.89] for nonvertebral fractures) (17). Zoledronic acid is superior to risedronate in BMD benefits (34). Teriparatide has robust evidence for its anti-fracture benefits (44), and appears to be even more effective in GIOP than primary osteoporosis in preventing vertebral fractures (69), perhaps due to GC effects on bone formation (59). Teriparatide also has evidence that it is more effective than alendronate, zoledronic acid, and risedronate in increasing BMD and preventing vertebral fractures (16, 44, 71). Denosumab, a monoclonal antibody with anti-resorptive effects, has superior effects on BMD over 24 months as compared to risedronate (67), though fracture rates and adverse effects were not statistically different between denosumab and risedronate. A comprehensive summary of evidence of the effectiveness of therapies for GIOP on fractures, BMD, and bone turnover has been recently published (18).

### Clinical Guideline Recommendations for Starting Treatment to Prevent GIOP: Who to Treat and Which Therapy

Current international clinical guidelines differ in their assessment of who is indicated for anti-GIOP therapy (the American College of Rheumatology [ACR, 2017] (72); the International Osteoporosis Foundation and European Calcified Tissue Society [IOF-ECTS, 2012] (73); Royal College of Physicians, National Osteoporosis Society, and Bone and Tooth Society [RCP, 2002] (74); and the UK National Osteoporosis Guideline Group [NOGG, 2017]) (75). A summary of recommendations from international clinical guidelines is presented in **Table 1**, with the ACR 2017 fracture risk criteria available in **Table 2**. Generally, all recommend that patients initiating ≥3 months of any dose of GC therapy and who have experienced a prior fragility fracture should start anti-osteoporosis therapy, regardless of age or GC dose. Similarly, for those without a prior fracture, fracture risk assessments that account for clinical risk factors (e.g., age, sex, GC dose) or estimate a GC-adjusted fracture probability (such as that derived from FRAX) are recommended to determine whether treatment is indicated. However, thresholds for treatment are heterogenous between guidelines, potentially from a lack of evidence examining the anti-fracture benefits of GIOP treatment among patients with varying fracture risk factors. Similarly, the guidelines differ in how patients are categorized as high versus low fracture risk. Nevertheless, all recommend starting therapy as soon as possible for those who are indicated based on evidence from pharmacologic and observational studies; however, only the ACR guidelines explicitly suggest undertaking a BMD measurement within three to six months of initiation as part of fracture risk assessment (72).

**Table 1:** Summary of guideline recommendations on anti-osteoporosis treatment for adults with glucocorticoid use.

Guideline	Populations to be treated with an anti-osteoporosis therapy	Treatment start	Treatment duration
**2017 American College of Rheumatology Guideline for the Prevention and Treatment of Glucocorticoid-Induced Osteoporosis **(72) (ACR 2017)	Adults aged ≥40 years at moderate risk** of fracture^C^	Fracture risk screening and potential treatment initiation as soon as possible, but max within 6 months of GC initiation for all patients with anticipated long-term GC treatment (≥3 mo)^X^	Adults ≥40 years continuing GC treatment: continue treatment as long as GCs used, then re-assess. Adults ≥40 years stopping GC treatment:
	Adults aged ≥40 years at high risk** of fracture^A^	Repeat fracture risk assessment every 12 months^X^	Low fracture risk: discontinue osteoporosis medication but continue calcium and vitamin D^C^ Moderate or high fracture risk: “complete” treatment with OP medication like for general osteoporosis^A^
	Adults age <40 years at moderate or high risk** of fracture^C^	Recommended first-line therapy: Oral bisphosphonates^C^	
	Special populations^C^:		
	Women of childbearing age at moderate to high fracture risk who do not plan to become pregnant within the period of OP treatment Adults aged ≥30 years receiving very-high dose GCs (initial dose prednisone ≥30 mg/day and cumulative dose > 5 g in 1 year) Adults with organ transplant, eGFR ≥30 ml/min and no evidence of metabolic bone disease who continue treatment with GCs		
**UK clinical guideline for the prevention and treatment of osteoporosis, National Osteoporosis Guideline Group, (NOGG, 2017) **(75)	Postmenopausal women taking GCs who are: over ≥70 years of age, have had a previous fragility fracture, or are taking ≥7.5 mg prednisolone/day or equivalent^C^	Start therapy immediately for indicated patients^C^	Continue treatment as long as GC use continues, can consider stopping if GC withdrawn^C^
	Bone protective therapy may be appropriate in some men and premenopausal women on GC therapy who have a previous fracture or are taking ≥7.5 mg/day prednisolone equivalent^C^	Suggested first-line therapy: Alendronate or risedronate^X^	
**International Osteoporosis Foundation and the European Calcified Tissue Society 2012 (IOF-ECTS 2012) **(73)	Postmenopausal women and men aged ≥50 years committed or exposed to ≥3 months oral GCs:	Start therapy at the onset of GC treatment^X^	Consider withdrawal of therapy with reassessment of fracture risk, preferably with a BMD measurement^X^
	≥70 years of age, or Prior fragility fracture, or taking ≥7.5 mg/day prednisone equivalent, or BMD T-score -1.5 or above country-specific GC-adjusted FRAX intervention threshold*^X^	Suggested first-line therapy: Bisphosphonates or teriparatide (choice of treatment mainly influenced by cost and tolerability)^X^	
	Premenopausal women and men <50 years committed or exposed to ≥3 months oral GCs who have had a prior fragility fracture^X^		
	Also consider treatment if taking ≥7.5 mg/day prednisone equivalent^X^		
**Royal College of Physicians, National Osteoporosis Society, and Bone and Tooth Society 2002 **(74)	Consider treating patients with anticipated GC use≥3 months ^C^: AND	Start at initiation of GC therapy^X^	Not specified
	>65 years, or prior fragility fracture, or BMD T score ≤-1.5^C^	No suggested first-line therapy.	

MOF, major osteoporotic fracture (clinical vertebral, hip, wrist, or humerus).

^A^recommendation based on randomized trial evidence.

^C^evidence based on expert opinion, pharmacologic/preclinical evidence, or first principles.

^X^recommendation not graded, evidence not assessed, or good practice recommendation only.

*thresholds derived locally due to limitations of the algorithm.

**See **Table 2** for definitions of high/moderate/and low fracture risk per the ACR 2017 guidelines.

**Table 2:** Fracture risk assessments in the 2017 American College of Rheumatology guideline for the prevention and treatment of glucocorticoid-induced osteoporosis.

High risk	Moderate risk	Low risk
Adults ≥40 years:	Adults ≥40 years:	Low risk:
Prior osteoporotic fracture, or Hip or spine BMD T score ≤-2.5 in men age ≥50 years or postmenopausal women, OR FRAX (GC-adjusted*) 10-year risk of MOF ≥20%, or FRAX (GC-adjusted*) 10-year risk of hip fracture ≥3% Adults <40 years: Prior osteoporotic fracture	FRAX (GC-adjusted*) 10-year risk of MOF 10-19%, OR FRAX (GC-adjusted*) 10-year risk of hip fracture >1% but <3% Adults <40 years: Hip or spine BMD T score < -3 or rapid bone loss (≥10% at the hip or spine over 1 year) AND Continuing GC treatment at ≥7.5 mg prednisone/day for ≥6 months	Adults ≥40 years:
		FRAX (GC-adjusted*) 10-year risk of MOF < 10%, OR FRAX (GC-adjusted*) 10-year risk of hip fracture ≤1% Adults <40 years: None of the above risk factors other than GC treatment

MOF, major osteoporotic fracture (clinical vertebral, hip, wrist, or humerus).

*If GC treatment >7.5 mg/day prednisone or equivalent, increase major osteoporotic risk by 1.15 (15%) and hip fracture risk by 1.2 (20%).

Despite general agreement that anti-osteoporosis medications should be initiated in patients at high fracture risk, the current guidelines do not consistently recommend certain therapies over others for GIOP patients. Only the ACR guideline explicitly recommends initiating oral bisphosphonates (in addition to calcium and vitamin D supplementation) over other anti-osteoporosis treatments (72). The IOF-ECTS and NOGG guidelines suggest that oral bisphosphonates can be considered for first-line therapy in GIOP patients (73, 75). The RCP guidelines do not suggest or recommend a certain therapy for GIOP (74).

Oral bisphosphonates are justified in the ACR guidelines as the preferred first-line therapy for the prevention of fractures in GIOP due to their robust effectiveness, oral formulation, low cost, well-characterized safety profile in immunosuppressed patients, and lack of evidence showing that other therapies have superior anti-fracture (not BMD) effectiveness (72). Zoledronic acid, denosumab, and teriparatide are recommended by ACR as second-line treatment if oral bisphosphonates are not effective or not tolerated (72). However, there is increasing clinical trial and observational evidence that teriparatide is superior to oral bisphosphonates in preventing vertebral fractures in GC-naïve and GIOP patients with severe spinal osteoporosis (44, 76, 77). Additionally, considering the pathophysiology of GIOP is driven by effects on osteoblasts, teriparatide is a particularly attractive treatment option as it stimulates bone formation (77). We therefore suggest that teriparatide may be considered as first-line therapy in patients at high risk of vertebral fractures (e.g., with a recent vertebral fracture or very low vertebral BMD). Teriparatide therapy is limited to 24 months in many jurisdictions and should be followed up with antiresorptive treatment (78). We also acknowledge that the use of teriparatide may be limited by cost, its required daily injections and its contraindications such as a prior history of skeletal malignancy or radiotherapy (79). Denosumab may not be a preferred first-line option for GIOP, as discontinuation is associated with a rapid loss of effectiveness and there is some limited data to suggest that fracture risk might be transiently increased (80–82). Anti-osteoporosis therapy is often stopped after GCs are discontinued, as discussed in the following section. Choice of treatment will likely also be influenced by clinical characteristics like menopausal status and renal dysfunction as well as insurance reimbursement and formulary policies, geographic location, costs, and patient preferences.

### Patient Case: Part 2 Response

Your patient is at high fracture risk and, despite having no prior fractures, we recommend that she be started on antiresorptive therapy at this visit. Our recommendation to treat is based on data that show independently increased risk of fractures for patients less than 50 years of age associated with: a) CD greater than 1350 mg (patient’s estimated cumulative dose at this timepoint: greater than 2700 mg) (24); b) a DD higher than 7.5 mg (11, 24) per day and the excess risk added when a patient has CD > 1 g *and >*15 mg DD per day (50); c) projected use longer than 3 months that can increase fracture risk even with low doses (11, 49); and d) a reduced BMD, which is also an important factor in determining her fracture risk (83). This recommendation is also in line with current recommendations from select guidelines: treatment is indicated due to GC exposure ≥ 30 mg prednisone per ACR guidelines (72). According to RCP guidelines (74), treatment is indicated because of projected therapy duration of more than 3 months with a DD of more than 7.5 mg/day in context of a BMD T-score which is less than -1.5. Current IOF-ECTS and NOGG guidelines, however, would not recommend starting treatment in this case (73, 75). MP would be indicated according to the IOF-ECTS guidelines if she had a prior fragility fracture *or* was older than 50 years of age and postmenopausal. Similarly, she would be indicated by the NOGG guidelines if she were postmenopausal, but these guidelines suggest that some premenopausal women taking ≥7.5 mg DD may be indicated for anti-osteoporosis therapy without further elaboration. Finally, we recommended using antiresorptive therapy (bisphosphonates) rather than teriparatide as first-line therapy, as your patient does not have severe spinal osteoporosis and thus antiresorptive therapy may be sufficient to prevent fractures. We would recommend an oral bisphosphonate as initial treatment due to the evidence of effectiveness for reducing fracture risk in GIOP (16), particularly with risedronate therapy; low cost, as highlighted by ACR recommendations (72); and ability for the clinician to stop treatment abruptly once bone protecting treatment is no longer required. However, the choice of bisphosphonate may be driven by cost, formulary restrictions, and patient/prescriber preferences.

## Stopping Treatment To Prevent GIOP

### Patient Case: Part 3

Nine months after disease onset, your patient’s joint symptoms have improved, mouth ulcers and pleuritic chest pain have resolved, and blood tests and urinalysis have normalized, indicating low SLE disease activity. The patient currently takes 7.5 mg prednisone each day, and you begin a tapering regimen for her prednisone over the next 12 weeks and continue HCQ and azathioprine therapy. She responds well to the tapering regimen and is found to have adequate adrenal reserve on 2.5 mg prednisone daily. Twelve months after her disease onset, the patient is able to stop prednisone altogether, and her disease remains well-controlled with combination treatment with HCQ and azathioprine.

Can you discontinue antiresorptive therapy? If yes, when should therapy be stopped?

### Fracture Risk Following GC Discontinuation

It is widely accepted that GIOP is to some extent reversible. Pre-clinical evidence suggests this reversibility comes from a rapid recovery of osteoblasts after GCs are discontinued. For example, in patients with Cushing’s disease, osteoblast activity as well as bone mineralization dramatically renews to baseline levels within 6 months after cure (84). GIOP’s effects on bone quality may endure beyond this period, however; patients with a median duration of remission from Cushing’s disease of 6 years showed similar BMD values as age- and sex-matched controls but had altered bone material properties (85). This impact on bone quality may come from longstanding impact of GCs on osteoclasts and osteocytes, which are less studied than osteoblasts (43).

While understanding recovery of preclinical markers helps to attest to the reversibility of GIOP, the duration of excess fracture risk after GC therapy is stopped determines the appropriate duration of anti-osteoporosis therapy. Although select studies have shown that prior GC use is associated with increased fracture risk (86) and FRAX includes previous use of GCs as a fracture risk factor (46), multiple large population-based studies have independently shown that fracture risk decreases rapidly after GC exposure is stopped (24, 51, 87). A 2018 US study of over 289,000 patients with GC use and chronic conditions (RA, asthma, chronic obstructive pulmonary disease, inflammatory bowel disease, multiple sclerosis, lupus, or sarcoidosis) found the first major decrease in fracture risk after 60 to 182 days off therapy (adjusted HR [versus current use] 0.73, a 27% decreased relative risk), with only marginal further decreases in risk after longer periods off-therapy (35% decreased relative risk after more than 365 days off therapy) (24). Similar trends were observed in a cohort of US patients with RA (87). An earlier study on intermittent glucocorticoid use examined the relationships between time since discontinuation, daily dose, cumulative dose and various types of fracture (51). Among those with >15 mg DD per day, a rapid decrease in the risk of all fractures was seen in the 3 months after discontinuation, with the most profound decreases observed for vertebral fracture risk in this period (51). No elevated risk was seen beyond 12 months after discontinuation of GC therapy. Those with less than 15 mg per day DD had no excess risk beyond 9 months after their last dose. When examining cumulative exposures, fracture risk returned to baseline levels after 6 months in those with less than 1 gram total CD, while those with ≥1 gram did not have a reduction to baseline levels until 15 months later. Conversely, a Danish population-based case-control study did not observe this dramatic decrease in vertebral fracture risk after discontinuation; when compared to never-users, distant past users (>1 year) still had an elevated risk of vertebral fracture (OR_adjusted_ 1.23 [1.16-1.30]) but no apparent risk of hip fracture (OR_adjusted_ 0.97 [95% CI 0.93-1.01]) (50). Notably, most studies have not examined whether fracture risk after GC discontinuation differs by age, sex, or other fracture risk factors.

Due to evidence of the reversibility of GC effects on bone, all current treatment guidelines suggest that anti-osteoporosis therapy can be stopped after GC is discontinued (**Table 1**). However, none recommend specific timing to stop therapy based on empiric evidence. In addition, most recognize that the role of BMD monitoring post-GC use has not been established. The highest quality evidence for lower fracture risk patients stopping anti-osteoporosis therapy after stopping GC treatment comes from observational studies (11, 24, 50, 51), though we note that some were published after the guidelines were released. Consequently, most of the guideline recommendations are based on expert opinion or *in vitro* studies as, to date, there are no trials examining fracture risk with varying durations of osteoporosis treatment after GC discontinuation. A recent review on the pharmacology of GIOP and GCs recommends continuing therapy for six to 12 months after discontinuation of GCs (43). The only recommendation that is strong with high quality evidence in the guidelines is the ACR 2017 recommendation to continue anti-osteoporosis treatment if the patient is indicated per primary osteoporosis guidelines after GC therapy, which is based on RCT evidence in primary osteoporosis (72).

Based on the available evidence, including preclinical data, stopping anti-osteoporosis treatment immediately at the time of GC discontinuation may not be ideal. However, additional real-world evidence on fracture risk following both GC and anti-osteoporosis medication is critical. In addition, estimating the exact end time of glucocorticoid exposure is difficult using many research data sources given tapering regimens and as needed doses that are not captured through claims data, so observational studies on the effects of discontinuing GCs and continuing anti-resorptive therapy beyond GC discontinuation often have methodological limitations (50). Nevertheless, based on epidemiologic studies of the duration of elevated fracture risk for up to 15 months after stopping GCs in some patients (24, 50, 51), clinicians might choose to continue therapy for another three to six months for lower cumulative exposures (e.g., less than 1 gram CD), with longer periods (e.g., six to eighteen months after GC discontinuation) for greater cumulative exposures.

### Patient Case: Part 3 Response

Based on existing clinical and preclinical evidence, since your patient is at low fracture risk aside from GC treatment, we recommend that antiresorptive therapy can be stopped after GC therapy is discontinued. We may consider continuing antiresorptive therapy for six months after discontinuation of GC therapy. The ACR, IOF-ECTS, and NOGG guidelines all recommend (ACR) or suggest (IOF-ECTS, NOGG) stopping anti-osteoporosis therapy once GC therapy is stopped (72, 73, 75). The RCP guidelines do not comment on discontinuing therapy once GCs are stopped (74). As stated in **Table 1**, no international guideline recommends a specific timing on stopping anti-osteoporosis therapy.

Our recommendation to stop anti-osteoporosis treatment and our suggestion to stop 6 months after last GC exposure are limited based on available evidence, particularly the effects of stopping among patients with high CD but are less than 50 years of age. First, evidence has shown that patients of similar age to MP (average age of 47 years) but who had cumulative exposures of 675 mg or less (substantially lower CD than MP) had a substantial decrease in fracture risk after 60 to 180 days of stopping GC therapy (24). Patients with CD greater than 1 gram, but who had an average age of 64 years also had a decrease in fracture risk starting at 3 months after last GC dose, but risk remained elevated from never users until 15 months after stopping GCs (51).

## Future Research

While there is abundant evidence that anti-resorptive and anabolic treatments help to prevent fractures in GIOP, there are clear gaps in knowledge regarding the timing of treatment, particularly when to discontinue in patients with few other fracture risk factors. Studies of the effects of discontinuation of GCs on fracture risk also have methodological limitations; future research could validate algorithms to ascertain true timing discontinuation of GC therapy to improve exposure measurement in fracture effects studies. In addition, most studies supporting the effectiveness were underpowered to study effects in subgroups with additional fracture risk factors (e.g., low body mass index, recent fragility fracture). Future studies in these subgroups are particularly important to determine the benefits of GIOP treatment in patients with different baseline fracture risk. In addition, some GCs may have bone-sparing effects (88) by controlling inflammation and providing better disease control (89). An RCT is underway to examine fracture and BMD outcomes among patients with RA randomized to low dose GC or placebo added to standard RA treatment that will provide evidence on this knowledge gap (90). The utility of microindentation measurements to assess and predict fracture risk, both while exposed to GC therapy and after discontinuation (85) is another future area of study. Finally, recent preclinical evidence suggests that GC-induced fracture risk might result in part from the disturbance in circadian rhythm (91), yet studies in humans are needed.

## Conclusion

Patients on long-term GC therapy should be assessed for fracture risk and potentially initiated on treatment to prevent GIOP. Most guidelines recommend initiating anti-osteoporosis therapy immediately for those on high-dose GC therapy, with a prior fracture, or at high fracture risk according to guideline-specific categories, though evidence shows other groups are also at risk of GC-induced fractures. Recommendations on stopping therapy with GC discontinuation are less clear. Though anti-osteoporosis therapy can be stopped in patients at low fracture risk after GC therapy is discontinued, it may be appropriate to continue therapy beyond GCs for a finite time (e.g., 6-12 months) due to a residual, dose-dependent fracture risk after stopping GC therapy. In particular, patients stopping after high cumulative GC exposure may benefit from extended treatment. Clinical trials comparing the relative anti-fracture benefits of varying lengths of treatment after GC discontinuation are critical to form strong recommendations on duration of treatment for GIOP.

## Author Contributions

KH drafted and revised this manuscript and provided final approval of this manuscript. EW contributed to conception, design, and interpretation of the work; revised this manuscript; and provided final approval of this manuscript. UB revised this manuscript; and provided final approval of this manuscript. BH contributed to conception, design, and interpretation of the work; revised this manuscript; and provided final approval of this manuscript. AB revised this manuscript; and provided final approval of this manuscript. All authors contributed to the article and approved the submitted version.

## Conflict of Interest

The authors declare that the research was conducted in the absence of any commercial or financial relationships that could be construed as a potential conflict of interest.

## Publisher’s Note

All claims expressed in this article are solely those of the authors and do not necessarily represent those of their affiliated organizations, or those of the publisher, the editors and the reviewers. Any product that may be evaluated in this article, or claim that may be made by its manufacturer, is not guaranteed or endorsed by the publisher.
