# Corrigendum: When to Start and Stop Bone-Protecting Medication for Preventing Glucocorticoid-Induced Osteoporosis

**DOI:** 10.3389/fendo.2022.877512

**Published:** 2022-03-14

**Authors:** Kaleen N. Hayes, Ulrike Baschant, Barbara Hauser, Andrea M. Burden, Elizabeth M. Winter

**Affiliations:** ^1^ Department of Health Services, Policy, and Practice, Brown University School of Public Health, Providence, RI, United States; ^2^ Division of Endocrinology, Diabetes and Bone Diseases, Department of Medicine III and Center for Healthy Aging, Technische Universität Dresden, Dresden, Germany; ^3^ Rheumatic Disease Unit, Western General Hospital, National Health Service (NHS) Lothian, Edinburgh, United Kingdom; ^4^ Rheumatology and Bone Disease Unit, Centre for Genomic and Experimental Medicine, Institute of Genetics and Molecular Medicine, University of Edinburgh, Edinburgh, United Kingdom; ^5^ Institute of Pharmaceutical Sciences, Department of Chemistry and Applied Biosciences, Swiss Federal Institute of Technology [Eidgenössische Technische Hochschule (ETH)] Zurich, Zurich, Switzerland; ^6^ Center for Bone Quality, Division of Endocrinology, Department of Internal Medicine, Leiden University Medical Center, Leiden, Netherlands

**Keywords:** glucocorticoid-induced osteoporosis, glucocorticoids, bone fractures, bone density, anti-resorptive treatment, bone density conservation agents, bisphosphonates, teriparatide

In the article as published originally, Elizabeth M. Winter was erroneously presented as the corresponding author instead of Ulrike Baschant.

The authors apologize for this error and state that this does not change the scientific conclusions of the article in any way. The original article has been updated.

## Publisher’s Note

All claims expressed in this article are solely those of the authors and do not necessarily represent those of their affiliated organizations, or those of the publisher, the editors and the reviewers. Any product that may be evaluated in this article, or claim that may be made by its manufacturer, is not guaranteed or endorsed by the publisher.

